# Association between Early Menopause, Gynecological Cancer, and Tobacco Smoking: A Cross-Sectional Study

**DOI:** 10.31557/APJCP.2021.22.10.3165

**Published:** 2021-10

**Authors:** Joyce Mary Kim, Yeun Soo Yang, Su Hyun Lee, Sun Ha Jee

**Affiliations:** 1 *Department of Public Health, The Graduate School of Yonsei University, Seoul, Korea. *; 2 *Institute for Health Promotion, Department of Epidemiology and Health Promotion, Graduate School of Public Health, Yonsei University, Seoul, Korea. *

**Keywords:** Early menopause, breast cancer, cervical cancer, tobacco, epidemiology

## Abstract

**Background:**

The rates of smoking among women are rising. Previous studies have shown that smoking is associated with early menopause. However, the association of gynecological cancer, including breast and cervical cancer, with early menopause and smoking, remains unclear. Therefore, this study aimed to determine the association between smoking and early menopause, breast cancer, and cervical cancer.

**Methods:**

This cross-sectional study used data from the Korean National Health and Nutritional Survey Examination (KHANES) (2016–2018). Early menopause was defined as menopause before 50 years of age.

**Results:**

A total of 4,481 participants were included in the analysis. There was no association between early menopause and cervical cancer (adjusted odds ratio [aOR]: 1.435, 95% confidence interval [CI]: 0.730–2.821), but women who had experienced early menopause had a significantly higher risk of breast cancer than women who had experienced normal menopause (aOR: 1.683, 95% CI: 1.089–2.602, p=0.019). Early menopause was not associated with an increased risk of breast cancer in ever-smoker (aOR: 0.475, 95% CI: 0.039–5.748), but was associated with a significantly increased risk of breast cancer in never-smokers (aOR: 1.828, 95% CI: 1.171–2.852).

**Conclusions:**

Early menopause was associated with an increased risk of breast cancer in women who had never smoked, but not in women who had ever smoked.

## Introduction

Menopause transition, is the time when the ovarian follicle pool falls below the standard threshold. It is a biological process experienced by all women (Tawfik et al., 2015). Natural menopause is described defined as a period of 12 or more months of amenorrhea that is not a result of specific medical procedures such as chemotherapy and surgery or other known causes (Tawfik et al., 2015). It is associated with a decline in female sex hormones, and affects the child-bearing process. While menopause occurs in all women, the age at which it occurs varies. 

Early onset of menopause is associated with an increased risk of various health conditions, such as certain types of cancer, heart disease, stroke, and osteoporosis, thus becoming, a serious public health issue, affecting women’s fertility rates worldwide (Shuster et al., 2010). Therefore, there is a need to evaluate the risk factors associated with early menopause in order to be able to inform women of the health risks. 

Several studies have been conducted regarding the risk factors associated with early menopause. A study by Mikkelsen et al., (2007) found that nulliparous women were at a higher risk of early menopause than parous women. Factors such as higher educational levels, moderate alcohol intake, and higher coffee intake were associated with a lower risk of early menopause. In contrast, they found a greater prevalence of pre-menopause among women who smoked during the perimenopausal age period, indicating that active smoking was a risk factor for early menopause. Further, they established dose-response effect of smoking on early menopause, and that active and passive smoking resulted in early menopause . 

The association between smoking and early menopause has been described as the decline in the quality and quantity of ovarian follicles caused by the byproducts found in cigarettes. Smoking causes fluctuation in the levels of reproductive hormones during the fertility period, which alters the follicular development patterns and consequently, leads to changes in the follicle-stimulating hormone levels. Further, the impact of smoke exposure on the follicle pool has been found to affect the timing of menopause (Tawfik et al., 2015). 

Prior studies have established that tobacco smoking has an adverse impact on hormone production in women, given that these toxins affect multiple sites involved in the hormonal synthesis. In this regard, considerable studies affirm the effects of active smoking on the timing of menopause. This aspect is also evident in the study given that a significant proportion of active and former smokers experienced early menopause (Hyland et al., 2015).

Prenatal smoke exposure has been found to lead to early menopause by damaging the follicles and suppressing their formation. A study by Lutterodt et al., (2009) found that prenatal smoke exposure damaged the somatic cells during the process of ovarian development, which could lead to early menopause. The higher risk of early menopause due to smoking makes women vulnerable to developing various health conditions such as gynecological cancers.

It is fundamental to note that menopause typically occurs between the ages of 45 to 55 years (Gold, 2011). For instance, Korean women have a 49.2-year average of menopause. However, this stage can occur earlier in life as a result of multiple factors. While some women dread menopause, others may find it relieving owing to the associated pain and other menstrual complications. In this regard, menopause symptoms vary from one woman to another, and they may be confused for other natural biological processes (Park et al., 2002). 

The current trend of increasing prevalence of smoking in women and the close connection between smoking and early menopause is a critical public health concern. While previous studies have addressed the association between smoking and early menopause , the association of gynecological cancer, including breast and cervical cancer, with early menopause and smoking, is not clear. Therefore, this study aimed to investigate the association of early menopause, breast cancer, and cervical cancer with smoking. 

## Materials and Methods

**Figure 1:**
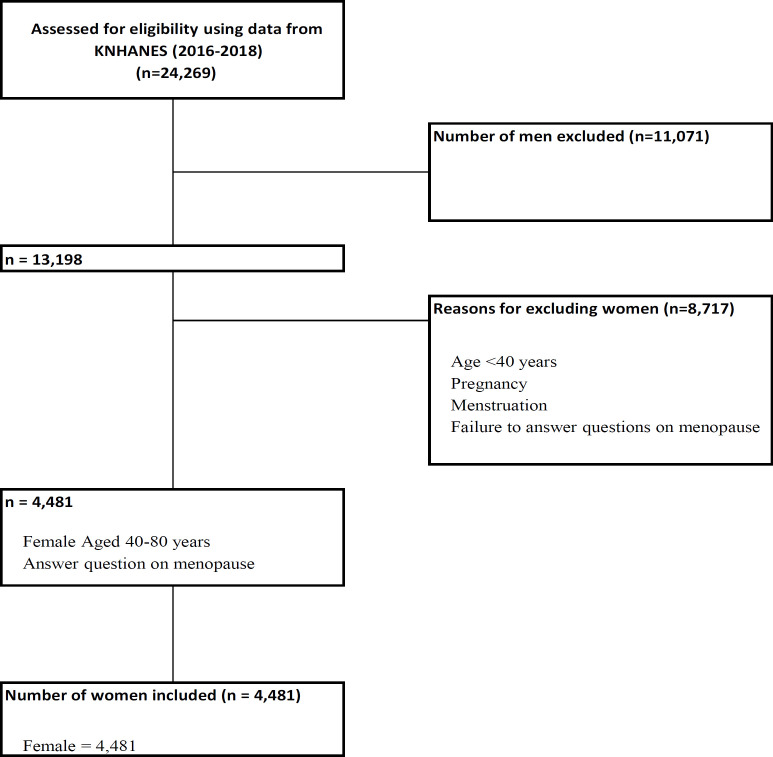
Flowchart of the Inclusion and Exclusion Criteria

This cross-sectional study used data from the 7^th ^editions of Korean National Health and Nutritional Survey Examination (KHANES), conducted from 2016 to 2018 by Korea Centers for Disease Control and Prevention, Ministry of Health and Welfare. The KNHANES is an annual cross-sectional survey, and its target population comprises nationally representative non-institutionalized South Korean citizens. The study population was screened for eligibility by KNHANES (Kweon et al., 2014). The gross population sample for this study, comprising 24,269 participants was selected between 2016 and 2018. Since this study was women-centric, 11,071 men were excluded from this study population leaving 13,198 women. Women aged < 40 years, and those who were pregnant or menstruating were excluded. Additionally, women who failed to answer the study questions regarding menopause, and those who provided responses such as ‘do not know,’ or had missing responses, were excluded. This resulted in the exclusion of an additional 8,717 participants from the study population. Women aged 40–80 years who were comfortable with answering questions on their menopause status were included. Finally, a total of 4,481 women were included in this analysis. Figure 1 presents a flow diagram of the study inclusion and exclusion criteria. In the logistic regression models, early menopause was defined as menopause that occurred before the age of 50 years, while normal menopause was defined as menopause that occurred at 50 years of age or above. In this study, the exposure variable was menopause (early menopause) resulting owing to smoking. The outcome of this exposure was gynecological cancer, which included cervical and breast cancers. Statistical analyses were performed using the SAS software, version 9.4 (SAS Institute).

The target population was first assessed for breast and cervical cancers. The variables considered were as follows: age (40–49, 50–59, and > 60 years), age at menopause (20–29, 30–39, 40–49, 50–59, and > 60 years), educational level (high school and college), household income (low, mid-low, mid-high, high), smoking status (current, sometimes, former, never, and do not know), drinking status (yes, no, do not know), body mass index (BMI) (underweight, normal, overweight), and exercise (yes, no). An analysis was conducted on the percentage of participants who experienced menopause at the various ages. Two models were used to calculate the odds ratio (OR) for breast cancer and cervical cancer based on the menopausal status. The first model was a crude model, which included the OR and 95% CIs for gynecological cancers with respect to the occurrence of menopause. The second model was an adjusted model, in which the ORs and 95% CIs for gynecological cancers with respect to the menopause status were adjusted for the variables in the study. The variables included in the second model were educational level, smoking status, drinking status, household income, BMI, and exercise. 

OR was used to evaluate the possibility of occurrence of breast and cervical cancer in women who had early menopause. It compares the odds of an event occurring after exposing the participant to specific risk factors with the odds of the same event in a controlled situation, where the participant is not exposed to any risk factor. In this study, OR enabled the understanding of the likelihood of developing cervical and breast cancers in women who experienced early menopause, and the possibility of cancer occurring in women who experienced menopause at the average age. Further, the OR enabled us to determine the relationship of early menopause, breast cancer, and cervical cancer with tobacco smoking in Korean women.

**Table 1:** General Characteristics of the Study Population

Variables	Total (N=4,481)"	Breast Cancer		Cervical Cancer	
		Yes	No	Yes	No
Age					
40-49	86 (1.92)	4 (5.56)	82 (1.87)	1 (2.86)	85 (1.91)
50-59	1,358 (30.31)	29 (34.12)	1,329 (30.23)	10 (28.57)	1,348 (30.32)
60=<	3,037 (67.78)	52 (61.18)	2,985 (67.90)	24 (68.57)	3,013 (67.77)
Menopause					
20-29	1 (0.02)	0 (0)	1 (0.02)	0 (0)	1 (0.02)
30-39	105 (2.34)	3 (3.53)	102 (2.32)	2 (5.71)	103 (2.32)
40-49	1796 (40.08)	43 (50.59)	1,753 (39.88)	17 (48.57)	1,779 (40.01)
50-59	2,547 (56.84)	39 (45.88)	2,508 (57.05)	16 (45.71)	2,531 (56.93)
60≤	32 (0.71)	0 (0.0)	32 (0.73)	0 (0.00)	32 (0.73)
Educational level*					
≤Highschool	3,892 (86.89)	65 (76.47)	3,827 (87.10)	31 (88.57)	3,861 (86.88)
≥College	587 (13.11)	20 (23.53)	567 (12.90)	4 (11.43)	583 (13.12)
Household Income**					
Low	1,513 (33.91)	24 (28.24)	1,489 (34.02)	18 (51.43)	1,495 (33.77)
Mid-low	1,149 (25.75)	22 (25.88)	1,127 (25.75)	11 (31.43)	1,138 (25.71)
Mid-high	910 (20.39)	20 (23.53)	890 (20.33)	3 (8.57)	907 (20.49)
High	890 (19.96)	19 (22.35)	871 (19.90)	3 (8.57)	887 (20.04)
Smoking Status					
Current	127 (2.83)	0 (0)	127 (2.89)	2 (5.71)	125 (2.81)
Sometimes	29 (0.65)	0 (0)	29 (0.66)	1 (2.86)	28 (0.63)
Former	157 (3.50)	3 (5.53)	154 (3.50)	1 (2.86)	156 (3.51)
Never	4,149 (92.59)	82 (96.47)	4,067 (92.52)	31 (88.57)	4118 (92.62)
Do Not know	19 (0.42)	0 (0)	19 (0.43)	0 (0)	19 (0.43)
Drinking					
Yes	1,208 (26.96)	26 (30.59)	1,182 (26.89)	12 (34.29)	1,196 (26.90)
No	3,259 (72.73)	59 (69.41)	3,200 (72.79)	23 (65.71)	3,236 (72.78)
Do Not know	14 (0.31)	0 (0)	14 (0.32)	0 (0)	14 (0.31)
BMI***					
Underweight(<23)	1,728 (38.80)	37 (43.53)	1,691 (38.70)	12 (34.29)	1,716 (38.83)
Normal(23~24.9)	1,088 (24.43)	21 (24.71)	1,067 (24.42)	13 (37.14)	1,075 (24.33)
Overweight(≥25)	1,638 (36.78)	27 (31.76)	1,611 (36.87)	10 (28.57)	1,628 (36.84)
Exercise					
Yes	2,463 (54.97)	43 (50.59)	2,420 (55.05)	20 (57.14)	2,443 (54.95)
No	2,018 (45.03)	42 (49.41)	1,976 (44.95)	15 (42.86)	2,003 (45.05)

*, Educational level -missing number N=2 (excluded); **, Household income - missing number N=19 (excluded); ***, BMI - missing number N=27 (excluded)

**Table 2:** Odds Ratios (95% Confidence Intervals) for the Risk of Gynecological Cancer According to Early Menopause Status

Early Menopause				
	(Unadjusted*) Model I		(Adjusted**) Model II	
	OR	CI	OR	CI
Cancer				
Breast Cancer	1.613	(1.048-2.481)	1.683	(1.089-2.602)
Cervical Cancer	1.615	(0.828-3.149)	1.435	(0.730-2.821)

Values are presented as Odd Ratio (95% confidence interval); *Model I, unadjusted; **Model II, was adjusted for education level, house income, drinking, BMI and exercise

**Table 3:** Odds Ratios (95% Confidence Intervals) of Early Menopause According to the Smoking Status

Early Menopause				
	(Unadjusted*) Model I		(Adjusted**) Model II	
	OR	CI	OR	CI
Non-Smokers				
Breast Cancer	1.695	(1.092-2.630)	1.828	(1.171-2.852)
Cervical Cancer	1.476	(0.728-2.992)	1.336	(0.653-2.734)
Ever-Smokersᵃ				
Breast Cancer	0.533	(0.048-5.943)	0.475	(0.039-5.748)
Cervical Cancer	3.263	(0.336-31.709)	4.545	(0.363-56.851)

Values are presented as odds ratio (95% confidence interval); *Model I, unadjusted; **Model II, was adjusted for educational level, household income, drinking habit, BMI, and exercise; ^a ^Includes current, sometimes, and former smokers.

## Results

Of the 4,841 study participants, 3,037 (67.78%), 1,358 (30.31%), and 86 (1.92%) were ≥ 60, 50-59, and 40-49 years old, respectively. Menopause was experienced by women across all age groups. As shown in Table 1, menopause was mostly experienced by the participants among ages 40-49 (n = 1,796; 40.08%) and 50-59 (n = 2,547; 56.84%). In contrast, it was least experienced by patients in the age-group 20-29 (n = 1; 0.02%).

Of the participants, 86.89% had a high school education and 13.11% had a college education. Most of the participants were from low- (33.91%) and mid-low-income (25.75%) families. Regarding the smoking status, 4,149 (92.59%) had never smoked, 127 (2.83%) were current smokers, 157 (3.50%) were former smokers, 29 (0.65%) were occasional smokers and 19 (0.42%) reported their smoking status as ‘do not know.’ Regarding alcohol consumption, 1,208 (26.96%) were current drinkers, 3,259 (72.72%) were non-drinkers, and 14 did not report their drinking status. For the BMI, 1,728 (38.8%) were underweight, 1,088 (24.43%) were of normal weight, and 1,638 (36.78%) were overweight. Additionally, a majority of the participants exercised (2,463, 54.97%). The distribution of gynecological cancers (cervical and breast) among the participants categorized according to the identified variables are summarized in Table 1.

Model 1, the crude model, revealed an increased likelihood of breast cancer and cervical cancer in women who experienced early menopause. ORs for breast and cervical cancers were 1.613 (95% CI: 1.048-2.481) and 1.615 (95% CI: 0.828-3.149), respectively, using normal menopause as the reference group. In contrast, there was a reduced possibility of cancer in women who experienced normal menopause. In Model 2, the adjusted model, the ORs were adjusted for educational levels, household income, exercise, BMI, and smoking and drinking habits. The adjusted ORs for breast and cervical cancer with respect to early menopause were 1.683 and 1.435, respectively. Additionally, the adjusted ORs for breast and cervical cancer among never-smokers were 1.828 (95% CI: 1.171–2.852) and 1.336 (95% CI: 0.653–2.734), respectively (Tables 2 and 3).

## Discussion

The variables considered in this research aimed to classify the study population according to their respective characteristics. This study focused on identifying how the selected variables affected the possibility of developing gynecological cancers (breast and cervical) in women experiencing early menopause. The ORs of breast cancer and cervical cancer according to the age at menopause were analyzed. 

The two models revealed a higher OR for breast cancer and cervical cancer according to the age at menopause. The first model indicated that early menopause increased the potential of breast cancer and cervical cancer in women. The second model suggested that early menopause was attributable to the study variables, namely household income, BMI, exercise, smoking and drinking habits, and education levels, which increased the possibility of women developing gynecological cancer. These findings were supported by Rosenberg et al., (2013) who found a close association between early menopause and the incidence of breast cancer. The study also showed that negative symptoms such as weight gain, fatigue, sleep challenges, and depression and anxiety, may characterize early menopause associated with the increased risk of breast cancer. 

According to Shuster et al., (2010), 25% of breast cancer cases involve premenopausal women and 75% involve menopausal women. Therefore, early menopause can be considered a risk factor for breast cancer. A study by Taneri et al., (2016) also found that natural menopause was associated with a reduced risk of breast, ovarian, and endometrial cancers. This indicated a decreased occurrence of breast cancer and cervical cancer in patients who experienced menopause at the normal age. 

The incidence rates of early menopause in women raise a serious public health concern (5%, early menopause; 1%, premature menopause) (Faubion et al., 2015). Thus, larger numbers of women are at risk of breast cancer and cervical cancer due to early menopause. Studies have proposed that coping with this challenge requires addressing the behavioral aspects associated with early menopause. Smoking is a modifiable and independent risk factor for early menopause. A study by Yang et al., (2015) on Korean women revealed that the menopausal age of current smokers was considerably lower than that of never-smokers. Therefore, addressing the challenge of smoking in women could significantly reduce the incidence of gynecological cancer. Moreover, according to Whitcomb et al., (2018), modifiable risk factors can have a significant impact on the aging of ovaries, and further, that smoking is also a lifestyle factor that can affect the timing of menopause significantly. In this study, the never-smoker status was significant, as the number of participants who had not smoked in their lifetime was low. The findings of this study have created a new area for future research which, when using more extensive data sets, should investigate smoking as an effect modifier. 

The major strength of this research was its large sample size that helps reduce statistical noise due to variations in participants’ characteristics (Andrade, 2020). This implies that the results of our research were authentic and reliable. Further, the large sample used in this research ensured that the study population is more representative of the entire Korean women population. Thus, the generalizations developed from this research can be used for policy formulation and further research. Furthermore, the study findings were consistent with those of past studies; hence, the findings have external validity. 

Our study also had limitations. First, it was not possible to verify the participants’ responses in the study. Therefore, the results may be affected by potential biases due to the possibility of incorrect answers. Further, this study did not extensively address the scope of smoking, particularly the association of passive smoking and early menopause with the occurrence of gynecological cancer. Passive smoking should have been considered a significant concern in this research since it affects many women who do not actively engage in smoking behavior. The non-inclusion of passive smoking in this research limits the scope of the study. More robust mathematical analyses using regression and correlation models should be applied to enhance the reliability of the research results. 

To conclude, the findings of this study suggested that smoking is positively correlated with a reduction in the menopausal age of Korean women. Moreover, this study identified an association between the occurrence of early menopause and gynecological cancers. Those who experienced menopause before the age of 50 years had (OR=1.613, 95% CI 1.048-2.481) of developing breast cancer than those who experienced menopause after the age of 50 years which was statistically significant. Additionally, early menopause and breast cancer were not associated in patients of the ever-smokers group, while those in the never-smokers group had (OR=1.695, 95% CI 1.092-2.630) of developing breast cancer. Thus, the association between early menopause and breast cancer is relevant, especially in non-smokers.

## Authors Contribution Statement

Joyce M. Kim, Yeun Soo Yang, Su Hyun Lee, and Sun Ha Jee contributed to the design and implementation of the research, to the analysis of the results and to the writing of the manuscript.
